# Practical guide for evaluation and management of beta-lactam allergy: position statement from the Canadian Society of Allergy and Clinical Immunology

**DOI:** 10.1186/s13223-020-00494-2

**Published:** 2020-11-10

**Authors:** Samira Jeimy, Moshe Ben-Shoshan, Elissa M. Abrams, Anne K. Ellis, Lori Connors, Tiffany Wong

**Affiliations:** 1 Division of Clinical Immunology and Allergy, Department of Medicine, Western University, London, ON N6A4V2 Canada; 2 Division of Allergy, Immunology and Dermatology, Department of Pediatrics, Montreal Children’s Hospital, McGill University, Montreal, QC Canada; 3 Department of Pediatrics, Section of Allergy and Clinical Immunology, University of Manitoba, Winnipeg, MB Canada; 4 Division of Allergy and Immunology, Department of Medicine, Queen’s University, Kingston, Canada; 5 Division of General Internal Medicine, Department of Medicine, Dalhousie University, Halifax, NS Canada; 6 Division of Allergy and Immunology, Department of Pediatrics, University of British Columbia, Vancouver, BC Canada

**Keywords:** Beta-lactam allergy, Antibiotic stewardship, Adverse drug reaction, Delabeling

## Abstract

The vast majority of individuals labelled as allergic are not deemed truly allergic upon appropriate assessment by an allergist. A label of beta-lactam allergy carries important risks for individual and public health. This article provides an overview of beta-lactam allergy, implications of erroneous beta-lactam allergy labels and the impact that can be provided by structured allergy assessment. We provide recommendations on how to stratify risk of beta-lactam allergy, beta lactam challenge protocols as well as management of patients at high risk of beta-lactam allergy.

## Background

Approximately 10% of the population carry a label of penicillin or beta-lactam allergy [1–3]. However, the vast majority of individuals labelled as allergic (up to 98%) are in fact beta-lactam tolerant upon appropriate assessment by an allergist [4–6]. A label of beta-lactam allergy carries important risks for individual and public health, as it is associated with increased use of second-line or broader-coverage antimicrobial treatments. These treatments may be of lesser efficacy and carry higher a risk of adverse outcomes, including longer hospitalizations, increased risks of antibiotic-resistant and Clostridium difficile infection, antimicrobial toxicity, and greater medical costs [7–9]. Herein we provide an updated review of the epidemiology and clinical spectrum of beta-lactam allergy. We provide a summary of negative implications of erroneous beta-lactam allergy labels on individual health and on the health care system, and the positive impact that can be provided by structured allergy assessment. We provide recommendations on how to stratify risk of beta-lactam allergy, based on clinical assessment. We provide recommendations on beta lactam challenge protocols. We also provide guidance for management of patients at high risk of beta-lactam allergy.

## Mechanisms and spectrum of beta-lactam allergy

The term “drug hypersensitivity” encompasses immune and non-specific adverse reactions to medications. “Drug allergy” is a subset of drug-hypersensitivity that refers to a specific immune response; the drug acts as a hapten, and the immune response is directed against a hapten-carrier complex that functions as the allergen. Several mechanisms leading to the immune response have been proposed, including the hapten model, pharmacologic interaction model and altered peptide repertoire model [(reviewed in [10]]. Non-immune mediated or “pseudoallergic” reactions has been attributed to plasma contact system activation [11].

Common phenotypes of beta lactam allergy can be classified according to the Gell and Coombs model, as outlined in Table 1 (Pichler AIM 2003). Most frequent reactions describe delayed-onset, morbilliform eruption, particularly in the pediatric population. The eruption may be caused by the infectious agent, usually viral (i.e., viral exanthem), or represent an immune response in the presence of a virus such as Epstein Barr virus (EBV) [5]. Immediate, IgE mediated reactions to beta-lactams are rare; as low as 5% of those who report an acute systemic reaction will have a reaction to an oral provocation challenge with penicillin, in some series [12]. In a retrospective chart review of 100 million people exposed to oral amoxicillin between 1972 and 2007 in the United Kingdom, there was one death in an adult patient due to anaphylaxis [13]. Delayed, T-cell-mediated reactions may be associated with specific HLA markers, including for flucloxacillin (HLA-B*57:01) and for amoxicillin-clavulanate (HLA-DRB1*15:01)-associated hepatitis [14]. However, the low positive predictive value (< 1%) of these HLA risk alleles and high number needed to test (NNT) to prevent 1 adverse reaction (> 10 000), makes HLA screening impractical for beta-lactam allergic reaction prevention.

**Table 1:** Clinical spectrum of beta-lactam hypersensitivity

Gell and Coombs Classification (extended)	Type of immune response	Pathologic characteristics	Clinical symptoms
Type I	IgE	Mast-cell degranulation	Urticaria, anaphylaxis
Type II	IgG and FC receptor	FCR-dependent cell destruction	Blood cell dyscrasia
Type III	IgG and complement or FC receptor	Immune complex deposition	Vasculitis
Type IVa	Th1 (Interferon-γ)	Monocyte activation	Eczema
Type IVb	Th2 (IL-5 and IL-4)	Eosinophilic inflammation	Maculopapular exanthema, bullous exanthema DRESS (drug reaction with eosinophilia and sytsemic symptoms)
Type IVc	Cytotoxic lymphocytes (perforin and granzyme B)	CD4- or CD8-mediated killing of cells	Macuolopapular exanthema, eczema, bullous exanthema, pustular exanthema SJS/TEN (Stevens Johnson Syndrome/Toxic Epidermal Necrolysis)
Type IVd	T cells (IL-8)	Neutrophil recruitment and activation	Pustular exanthema

Adapted from ref. [55]

*IL *Interleukin, *Th*  T helper

A noteworthy presentation of a beta lactam associated reaction is the serum sickness like reaction (SSLR). SSLR is defined as an immunological condition characterized by skin rash and arthralgia, with or without fever. Skin lesions are characterized by fixed erythematous and edematous patches/plaques and annular lesions with central clearing and/or purplish discoloration. The symptoms can present several days to several weeks after exposure of the trigger. In addition to the characteristic cutaneous manifestations, patients with SSLRs are reported to have malaise, lymphadenopathy, abdominal pain, nausea, vomiting, diarrhea, myalgias, headaches and a self-limited symmetric arthritis. SSLR is different than classic serum sickness as it is not associated with antigen–antibody complex formation and the blood levels of complement are usually normal. Further, in contrast to true serum sickness, renal and hepatic involvement is rare [15]. Although it is frequently recommended to avoid the triggering medication, there is increasing evidence that a hallmark of SSLR is the benign outcome and that future courses of the medication are unlikely to lead to recurrence [16, 17].

Based on the wide clinical spectrum and misunderstanding around adverse reactions to beta-lactams, a detailed clinical history is essential to the assessment of possible allergy, and for driving investigations and management. The authors propose a clinical history template in Table 2.

**Table 2:** Important clinical questions to clarify a beta-lactam adverse reaction history

1. When did the reaction occur?
2. Which medication was prescribed, and what was the route of administration?
3. What was the indication for the medication?
4. How many courses of this medication or a related medication have been administered?
5. How many doses were received prior to onset of reaction?
6. How soon after the most recent dose did the reaction occur?
7. Were there any concurrent medications administered?
8. What was the nature of the reaction? Specifically ask about:
Raised, erythematous, pruritic rash with each lesion typically lasting less than 24 h? (hives/urticaria)
Swelling of the tongue, mouth, lips, or eyes (angioedema)
Respiratory or hemodynamic changes (anaphylaxis)
Lesions or ulcers involving the mouth, lips, or eyes; skin desquamation (Stevens Johnson Syndrome (SJS), Toxic Epidermal Necrolysis (TEN), and other severe type IV reactions)
Organ involvement such as hematologic, renal, or hepatic (cytopenias, Acute Interstitial Nephritis (AIN), transaminitis)
Drug Reaction with Eosinophilia and Systemic Symptoms (DRESS) syndrome, and other severe type IV reactions)
Joint pain (serum-sickness like reaction)
Rashes that were not hives, were mild, or delayed in onset (mild type IV reaction or morbilliform rash)
Nausea, vomiting, diarrhea, minor laboratory abnormalities or local injection reactions
9. Was the medication stopped?
10. Was medical attention sought in an emergency room or from a community physician?
11. How was the reaction managed?
12. Were there symptoms of unexplained fever, arthritis/arthralgia, lymphadenopathy, skin exfoliation or mucous membrane involvement?
13. How long did symptoms last?
14. Were any symptoms consistent with a severe cutaneous adverse drug reaction (e.g., SJS, DRESS or AGEP)?
15. Has the same medication been taken subsequently? If yes, was there a reaction?

Adapted from [26] and [48]

## Beta-lactam structure and cross-reactivities

Beta lactam medications share a core ring, and structural differences between them are conferred based on adjacent rings and R-group side chains (Table 3). Beta-lactams belonging to the penicillin-class have an R1 side chain only. This R1 side chain is shared between some penicillins and cephalosporins, as well as among cephalosporins, and is thought to contribute to cross-reactivity. Earlier studies quoting 10% cross-reactivity between penicillins and cephalosporins are now known to be primarily caused by contamination of the early cephalosporin preparations with penicillins [18]. At present, 2% of patients with positive reactions to multiple penicillin skin-test reagents have demonstrated sensitization to cephalosporins [19–21]. Cefazolin has a unique side chain and very low cross-reactivity with penicillin [22]. There is no immunologic or clinical cross-reactivity between penicillins and the monobactam aztreonam; however, in patients who are allergic to ceftazidime, there have been reports of aztreonam reactions, which is due to a shared R1 side chain [20, 23] (Table 3).

**Table 3:**
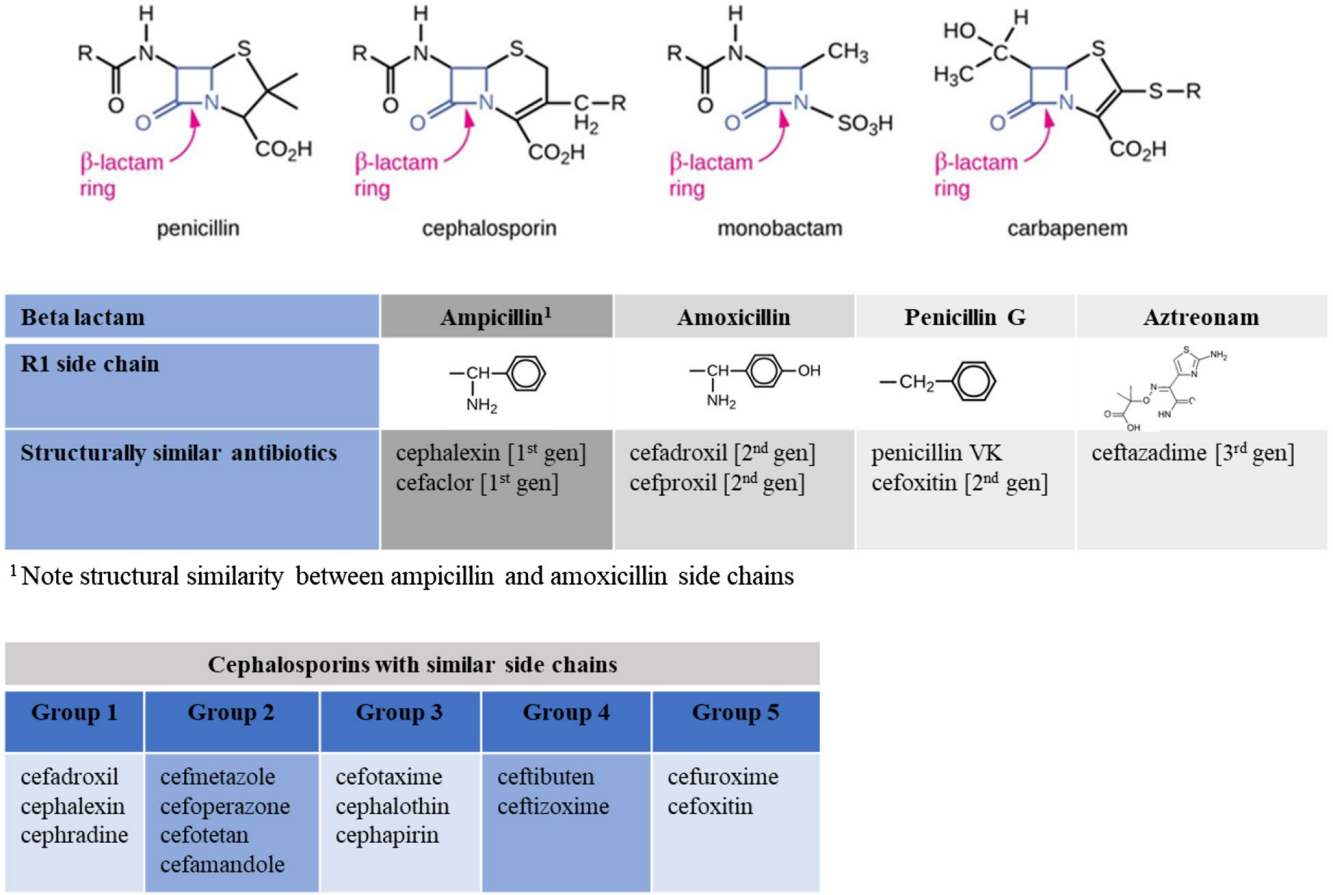
Probable beta-lactam cross-reactivities based on side chain similarities

Adapted from [19] and [49]

## Clinical implications of erroneous labels of beta-lactam allergy

The public health implications of erroneous labelling of beta-lactam allergy are well established, including an increased length and cost of hospital stay, decreased infection resolution rates, increased risk of infection recurrence, increased risk of adverse events from use of second-line antibiotics including C-difficile infections, and even increased mortality [10, 11, 24–26]. The over-labelling of beta-lactam allergy is associated with increased costs and antimicrobial resistance; testing for beta-lactam allergy would be 9.5 times less expensive than treating an in-patient population with an alternative antimicrobial [5].

For these reasons, removal the penicillin allergy label (“delabeling”) is recommended by multiple organizations, including the American Academy of Allergy, Asthma, and Immunology [27], the Infectious Disease Society of America [28], Canadian Pediatric Society [29], l’Institut National d’Excellence en Santé et en Services Sociaux (INESSS) in Quebec [30], Choosing Wisely Canada [31], and the Centers for Disease Control and Prevention [32].

## Diagnostic tests for beta-lactam allergy

Beta-lactam allergy assessment tools can include a clinical history, skin testing, and the gold standard of provocation challenge. Antigens used for skin testing include the major antigenic determinant of penicillin (PPL, benzyl penicillin), and the minor-antigenic determinant mixture. Although skin tests for penicillin have a quoted high negative predictive value (93%) based on a 1971 study in adults, recent studies have shown that sensitivity in the pediatric population can be as low as < 10% [5, 33]. As well, the positive predictive value of skin testing is poor, ranging between 50 and 75% [33], making skin testing a poor screening tool. In one retrospective review, patients with a nebulous history of penicillin allergy had rates of skin sensitization identical to that of patients without a penicillin allergy history (1.7%) [34]. Although a positive penicillin skin test result is often considered the reference standard for penicillin allergy, some patients may be sensitized (i.e., positive skin test result) but not clinically allergic, as shown by a negative oral penicillin challenge [35, 36]. Standardized skin tests for cephalosporins are not available, although non-irritating concentrations for skin-testing have been published [37].

**Fig. 1:**
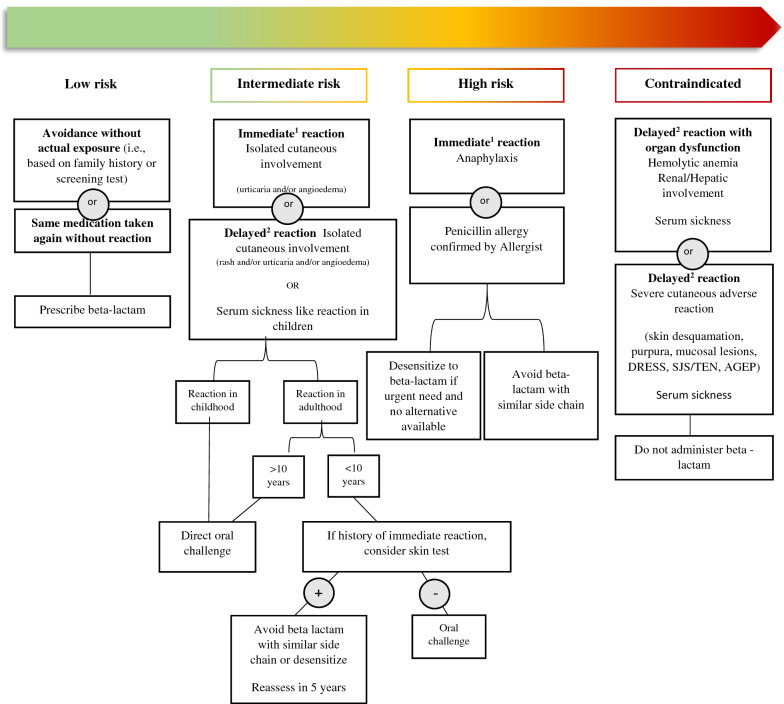
Penicillin allergy risk stratification, and contraindications to re-administration. 1. Immediate reaction (type I or IgE-mediated): symptoms within 2 h after taking the first dose, and duration of symptoms < 24 h. 2. Delayed reaction (types II, III and IV): symptoms > 2 h after drug administration

Although serum specific immunoglobulin E (sIgE) testing for beta-lactams is available, it also has poor positive and negative predictive value, and can identify clinically irrelevant co-reactivity between beta-lactams [38, 39]. The presence of measurable anti-beta-lactam IgE does not necessitate that exposure will result in an allergic reaction. Serum specific IgE testing for beta-lactam allergy, therefore, are suboptimal screening measures and not recommended. Oral provocation challenge is the gold standard diagnostic test for beta-lactam allergy. Based on clinical history, patients can be stratified into low, moderate, or high risk of adverse reaction. In low-risk patients (Fig. 1), proceeding directly to a single-step or graded oralprovocation challenge is a reasonable option. The graded oral provocation challenge consists of administering 10% of a therapeutic dose. In the absence of symptoms, after 30–60 min, the remaining 90% of the therapeutic dose is given. This is followed by a minimum 60-min period of observation [5, 40]. In intermediate risk patients, penicillin skin testing may be considered before the challenge. For patients with a history of penicillin allergy only, all beta-lactams can be administered as indicated after amoxicillin challenge is successful.

Alternate methods for beta-lactam allergy assessment, including patch testing, delayed intradermal skin testing, in vitro lymphocyte transformation assays and basophil activity testing are not well established, and they are not to be routinely recommended for routine assessment of any category of beta-lactam allergy at this time [41–44].

## Direct challenge without skin testing

Beta-lactam skin testing has selective availability, is not always feasible, and can have poor test characteristics, with a false-positive test rate of up to 80% [45]. There is a growing body of evidence demonstrating that direct challenges without skin testing is safe, and a preferred diagnostic approach in children with a history of mild cutaneous exanthems and adults with remote (> 5 years ago) history of mild symptoms not suggestive of anaphylaxis or severe cutaneous/systemic adverse reaction. This has led to a paradigm shift in beta-lactam allergy management. This recommendation is based on several studies, including a Canadian observational study of 818 children with a history of cutaneous or low risk reactions to amoxicillin; on amoxicillin challenge, 2.1% of the children had immediate reactions, and 3.8% had non-immediate mild reactions [5]. Of note, all of the immediate reactions were mild and entailed urticaria in the absence of other symptoms to meet criteria for anaphylaxis [5]. This study corroborates others, including a retrospective cohort study of 369 children who had a small number (3.8%) of mild reactions on amoxicillin challenge [46]. Of note, these children had negative penicillin skin tests. In another prospective, multicenter study of 732 children, 0.8% of the children had immediate reactions (1 required epinephrine administration), and 4% had delayed exanthems after the challenge [45]. Taken together, these studies add to the growing body of evidence that direct oral provocation challenge is a reasonable means of diagnosing beta-lactam allergy in the pediatric population. Despite this mounting data, skin testing may be considered in the context of patient/caregiver hesitancy to proceed with an oral provocation challenge in the absence of a skin test.

The direct oral provocation challenge is also indicated in other selected populations with a low pre-test probability of clinically significant reaction. These patients include those with a nebulous or remote (reaction more than ten years ago) clinical history [47, 48]. For patients who have had adverse reactions suggestive of intolerance rather than allergy (i.e., nausea, vomiting, or diarrhea), or have no personal history of reaction, but avoid beta-lactams because of a family history or positive screening tests, there is no increased risk of beta-lactam allergy beyond baseline and the medication can simply be prescribed.

Most reactions to beta-lactams during challenge entail minor symptoms, such as mild, self-limited, cutaneous eruptions. Patients can also report subjective symptoms including nausea or pruritus; in the absence of objective findings, these reactions are unlikely to be due to an allergy and can be monitored. In the case of cutaneous reactions, monitoring along with treatment with non-sedating, second generation antihistamines are suggested. If there are systemic symptoms, including generalized urticaria, or any symptoms that meet World Allergy Association (WAO) anaphylaxis criteria, the challenge should stop, and anaphylaxis management promptly initiated [49]. For this reason, it is important to have appropriate supplies for anaphylaxis management, including the ability to promptly administer epinephrine [49], available at the time of oral provocation challenge. A proposed oral provocation challenge protocol, along with guidelines for clinical assessments and management of symptoms, is provided in Table 4. The challenge can be performed in a graded manner, or in one-step, based on the pre-test probability of a clinical reaction in the patient.

**Table 4:** Graded challenge—suggested protocol and medications to have on-hand

Medications to have on-hand		
Medication	Dose	
	Pediatric	Adult
Epinephrine 1 mg/mL (1: 1000) administered IM	0.01 mg/kg > 25 kg: use Adult dosing	0.5 mg
Antihistamine	Diphenhydramine if unable to tolerate oral medications 1 to 2 mg/kg/dose (IM or PO); maximum: 50 mg/dose	25–50 mg
	Cetirizine 6 m to < 2 years: 2.5 mg 2 to 5 years: 2.5–5 mg ≥ 6 years: use adult dosing	10–20 mg
Glucocorticoids	Prednisone 1–2 mg/kg	20–60 mg
Short-acting bronchodilator (metered dose inhaler with spacer)	4–8 inhalations every 20 min for 3 doses	4–8 inhalations every 20 min for up to 4 h

## Follow up of patients after a negative challenge

Once a patient tolerates an oral provocation challenge with a beta-lactam, the patient should be counselled accordingly, that they do not have an increased risk of adverse reaction beyond that in the baseline population. The most common reason that patients reacquire a beta-lactam allergy label after negative challenge result is due to incomplete removal of the allergy label in various aspects of medical records existing in different locations [50]. Electronic health records are used widely in hospitals and ambulatory settings; it is important to update the patient’s health records to remove the notation that a patient is allergic. In the pediatric population, up to 10% of patients with negative oral provocation challenge may present with a mild cutaneous exanthem on subsequent beta lactam exposure; this exanthem is not a contraindication to treatment [5]. Updated medication allergy documentation should be communicated to all providers in the patient’s circle of care, including primary care physicians, pharmacists, and other caregivers’ records should be updated.

Written instructions to re-iterate that a patient had successful challenge, as well as a reminder of the symptoms of anaphylaxis, and when to report to an emergency department, in case of a severe reaction, can be helpful. Proposed written instructions to provide to a patient after a successful challenge are provided in Table 5. Allergists can provide a wallet card, confirming that the beta-lactam allergy was assessed, and the patient was found not to be allergic. It is important to discuss with the patient that after a challenge, they may experience delayed onset, benign rash, at an incidence similar to that of the general population. Longer oral provocation challenges to exclude delayed rashes is not recommended based on prolonged and unnecessary exposure to antibiotics, as well as poor diagnostic yield [51].

**Table 5:**
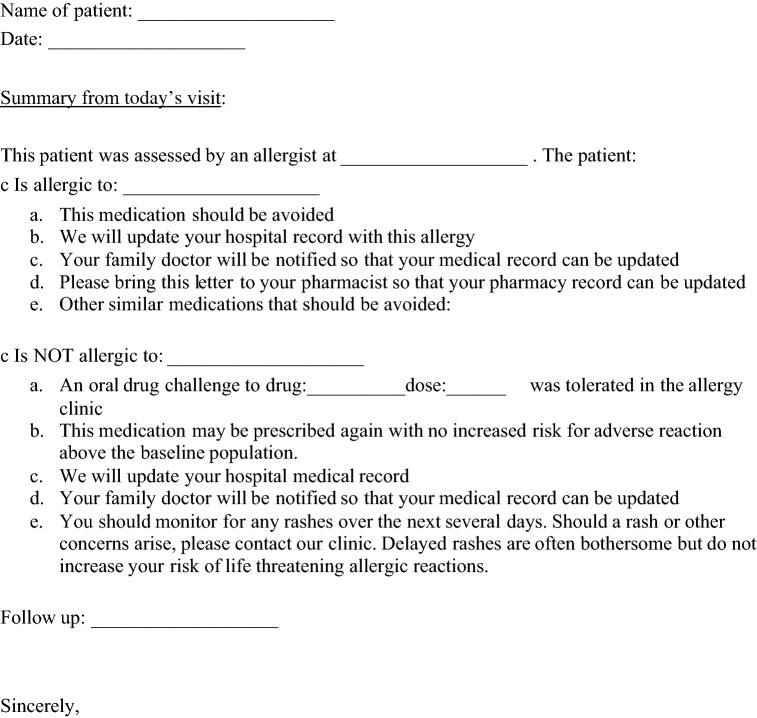
Proposed clinic letter for patients after provocation challenge to evaluate of beta lactam allergy

## Alternative antibiotic selection in the setting of confirmed beta-lactam allergy

For individuals with suspected IgE-mediated allergy, cephalosporins with similar side chains should be avoided. Cephalosporin medications with dissimilar side chains can be prescribed [52]. Patients allergic to beta-lactams (with the exception of ceftazidime) may safely receive aztreonam. There is very low clinical cross reactivity between carbapenems and beta-lactams, and beta-lactam allergic patients may receive graded drug challenges of carbapenemsn [53]. In patients with established amoxicillin allergy, cefixime has been well tolerated [5].

Individuals who have experienced severe systemic or cutaneous delayed adverse reactions following a dose of penicillin, should not be prescribed this antibiotic in the future There is no robust evidence to indicate cross-reactivity between specific penicillins or penicillins and cephalosporins with similar side chains in severe delayed allergic reactions. Future decisions for penicillin use other than the ones implicated should be based on benefit versus risk assessment on a case-by-case basis. Some organizations recommend avoiding cephalosporins with similar side chains in such cases [54].

## Desensitization in the setting of IgE mediated beta-lactam allergy

Desensitization may be indicated in several circumstances, including immediate need for a specific beta-lactam with no available testing or high suspicion of past IgE-mediated reaction, and inability to tolerate potential anaphylaxis due to low cardiorespiratory reserve or other factors. Desensitization to beta-lactams involves administration of increasing incremental doses of the drug performed under close clinical monitoring and co-ordination with pharmacy services (protocol outlined in [6]). The process is resource intensive and can potentially delay initiation of appropriate therapy; therefore, desensitization should be used judiciously in the appropriate patient, at high-risk of anaphylaxis on beta-lactam exposure. It is important to note that once a patient undergoes desensitization, regular and uninterrupted exposure to the medication is required to prevent re-sensitization.

## Conclusions

The label of beta-lactam allergy is common, affecting 10% of the population, and carries with it a risk of negative clinical and socioeconomic outcomes, including use of less desirable alternative antibiotics, longer hospitalizations, increased rates of antibiotic-resistant infections, and greater medical costs. Among patients who report a penicillin allergy, up to 98% have negative testing and can safely receive that antibiotic in the future. Patients can be erroneously labeled with a beta-lactam allergy due to misclassification of the suspected reaction. Patients with a suspected allergy to beta-lactam are often not referred to an allergist for evaluation and are instead prescribed alternate antimicrobials that may be less effective, have more side effects, or are more expensive. Recent studies suggest that risk stratification of patients prior to testing or challenge, and in most circumstances proceeding directly to oral provocation challenge, is safe and preferred.

## Data Availability

All references can be found online in medical literature databases. We do not present any previously unpublished data.

## Abbreviations

EBV: Epstein Barr virus
HLA: Human leukocyte antigen
PPL: Penicilloyl polylysine (PPL)
SCAR: Severe cutaneous adverse reactions
sIgE: Serum immunoglobulin E
TH2: Type 2 helper T cells
